# Deep learning–based automated detection of oral squamous cell carcinoma in histopathological images: a comparative study of five CNN architectures

**DOI:** 10.1007/s10266-026-01448-7

**Published:** 2026-06-15

**Authors:** Yunus Balel, Kaan Sağtaş, Fatih Teke, Mehmet Ali Kurt

**Affiliations:** 1 Department of Oral and Maxillofacial Surgery, Faculty of Dentistry, Sivas Cumhuriyet University, Sivas, Turkey; 2 SEMRUK Technology Inc, Cumhuriyet Teknokent, Sivas, Turkey; 3 Department of Industrial Engineering, Sivas Cumhuriyet University, Sivas, Turkey; 4 Department of Computer Engineering, Yildiz Technical University, Istanbul, Turkey; 5 Department of Machine Engineering, Sivas Cumhuriyet University, Sivas, Turkey

**Keywords:** Oral squamous cell carcinoma, Deep learning, Histopathological image analysis, Convolutional neural networks, Digital pathology

## Abstract

Oral squamous cell carcinoma (OSCC) is the most common malignancy of the oral cavity, and early diagnosis plays a crucial role in improving patient prognosis and survival rates. Histopathological examination remains the gold standard for OSCC diagnosis; however, this process is time-consuming and highly dependent on expert interpretation. With the rapid development of digital pathology and artificial intelligence, deep learning–based approaches have emerged as promising tools to support automated diagnostic systems. In this study, five convolutional neural network (CNN) architectures—VGG16, ResNet50, InceptionV3, EfficientNetV2S, and ConvNeXt-Tiny—were comparatively evaluated for the automated classification of OSCC using histopathological images. An open-access OSCC dataset was utilized, and two experimental scenarios were created using the original dataset and an augmented dataset. The dataset was divided into training, validation, and test subsets using a stratified approach. All models were trained under identical experimental conditions using ImageNet-pretrained weights and a unified classifier head in order to ensure a fair comparison. Model performance was assessed using Accuracy, Precision, Recall, Specificity, F1-Score, and ROC-AUC metrics. Additionally, Grad-CAM was applied to visualize the image regions influencing model predictions and to enhance interpretability. The results demonstrated that data augmentation significantly improved the performance of all models. Among the evaluated architectures, ResNet50 achieved the highest diagnostic performance on the augmented dataset, reaching an accuracy of 0.91 and a ROC-AUC of 0.88, followed by EfficientNetV2S and ConvNeXt-Tiny. Visualization analyses indicated that the models focused on histopathologically relevant regions associated with tumoral structures. Overall, the findings suggest that deep learning–based approaches can effectively support patch-level automated OSCC classification from histopathological images and may contribute to future development of clinical decision support systems in digital pathology.

**Supplementary Information:**

The online version contains supplementary material available at https://doi.org/10.1007/s10266-026-01448-7.

## Introduction

Oral Squamous Cell Carcinoma (OSCC) is one of the most common cancers in the head and neck region and accounts for approximately 90% of oral cavity malignancies [1]. Globally, hundreds of thousands of new cases are reported each year, and mortality rates remain relatively high [2]. The prognosis of OSCC largely depends on the stage at which the disease is diagnosed; survival rates are significantly higher in patients diagnosed at an early stage, whereas they decrease considerably in advanced stages [3]. Therefore, early and accurate diagnosis plays a critical role in the management of the disease. However, in current clinical practice, the diagnostic process largely relies on histopathological examination, and this process may vary depending on the experience of expert pathologists [4]. 

The evaluation of histopathological images is a process that requires a high level of expertise, is time-consuming, and contains a certain degree of subjectivity. The increasing patient workload and the limited number of specialists have increased the need for the automation of diagnostic processes. In this context, with the development of digital pathology, it has become possible to obtain high-resolution histological images, and the use of computer-assisted methods for analyzing these data has become widespread [5]. In particular, deep learning approaches have achieved significant success in medical image analysis due to their ability to automatically extract meaningful features from complex image data [6, 7]. 

Convolutional Neural Networks (CNNs) are widely used in critical fields such as cancer diagnosis due to their high performance in image classification tasks. In the literature, architectures such as VGGNet, ResNet, and Inception have enabled important advances in the field of image processing; more recently, next-generation architectures such as EfficientNet and ConvNeXt have demonstrated remarkable results in terms of accuracy and computational efficiency [8–12]. These developments make it possible to perform automatic disease diagnosis using histopathological images and contribute to the development of clinical decision support systems.

Nevertheless, studies comparing the performance of different CNN architectures on histopathological images of OSCC are limited, and existing studies generally focus on a single model or on narrow-scope comparisons [13, 14]. In particular, the systematic comparison of classical and next-generation architectures under the same dataset and experimental conditions has not been sufficiently addressed in the literature. This situation makes it difficult to conduct a clear evaluation of which model is more suitable for clinical applications.

In this study, five different CNN architectures (VGG16, ResNet50, InceptionV3, EfficientNetV2S, and ConvNeXt-Tiny) were comparatively investigated to perform automatic OSCC diagnosis from histopathological images. The main objective of the study is to evaluate the classification performance of these architectures using various metrics and to determine the most suitable approach for OSCC diagnosis. In this context, the hypothesis that deeper and more optimized modern architectures will demonstrate higher performance compared to classical models is tested. The findings are expected to both fill an existing gap in the literature and contribute to the development of clinical decision support systems.

Despite the growing number of studies applying deep learning to histopathological images of OSCC, several methodological limitations remain. Many existing studies focus on a single architecture, use heterogeneous experimental settings, or do not ensure strict separation between training and evaluation data, making it difficult to draw reliable conclusions about model performance.

The present study addresses these gaps by providing a systematic and controlled comparison of five widely used CNN architectures under a unified experimental framework. All models were trained using the same dataset, identical preprocessing and training configurations, and a shared classifier head to ensure a fair comparison. In addition, the effect of data augmentation was evaluated under a fixed and independent test set, allowing a more reliable assessment of its true impact on model performance.

Furthermore, the dataset structure ensured that each image corresponded to a distinct patient, eliminating the risk of data leakage and strengthening the validity of the results. By combining methodological rigor with a comprehensive multi-architecture comparison, this study provides clearer insights into the relative performance and practical applicability of deep learning models for OSCC diagnosis.

The novelty of this study lies not only in the comparison of multiple CNN architectures, but also in the use of a rigorously controlled experimental design. Unlike many previous studies employing heterogeneous preprocessing pipelines, variable classifier structures, or potentially overlapping datasets, all models in the present study were evaluated under identical preprocessing, augmentation, training, and testing conditions using a leakage-free dataset structure. Furthermore, both classical and next-generation CNN architectures were systematically compared within the same framework, providing a more reliable assessment of their relative performance and practical applicability for automated OSCC diagnosis.

## Materials and methods

Since an open-access histopathological dataset was used for artificial intelligence model training in this study, it is exempt from ethics committee approval [15]. The diagnoses of normal oral tissue and oral tissue containing OSCC in the histopathological images in the dataset were re-evaluated and confirmed by an oral and maxillofacial surgeon and dentist, who are authors of this study and experienced in oral pathology.

### Dataset

For the training of the artificial intelligence model, the OSCC histopathological image dataset published by Rahman et al. was used [15]. According to the dataset description provided by Rahman et al., each histopathological image corresponds to a distinct specimen obtained from a different patient, and no duplicated patient samples are included in the dataset [15]. In addition to relying on the original dataset documentation, we carefully inspected the dataset organization, file structure, image naming patterns, and metadata provided by the authors to identify any potential duplicated or related samples. No evidence suggesting repeated patient-derived images or overlapping specimens was identified during this verification process. Based on both the original dataset description and our independent dataset inspection, the dataset was considered suitable for leakage-free experimental design and patient-independent dataset splitting.

In order to evaluate the generalizability of deep learning models and to control the overfitting condition, the dataset was divided into three parts using a stratified approach. To ensure the reproducibility of the algorithms, the random seed was fixed at 58. 80% of the dataset was allocated for training, 10% for validation, and 10% for testing.

To prevent data leakage, special attention was given to the independence of samples during dataset splitting. Each histopathological image in the dataset represents a distinct specimen obtained from a different patient. Therefore, there are no multiple images originating from the same patient or slide. Additionally, manual inspection of the dataset metadata and file organization was performed to further confirm the absence of duplicated or related patient samples across subsets.

Based on this structure, the dataset was split at the image level using a stratified approach; however, since each image corresponds to a unique patient, this effectively ensures patient-level separation between the training, validation, and test sets.

Furthermore, a fixed random seed (58) was used during the splitting process to ensure reproducibility, and the same split was consistently applied across all experiments. As a result, no related or overlapping samples were present across different subsets, effectively eliminating the risk of data leakage.

### Preprocessing

All images were resized to a fixed input size compatible with each backbone architecture. Pixel values were normalized using standard ImageNet normalization parameters to match the pretrained weights. No additional stain normalization or color standardization techniques were applied.

### Data augmentation

To improve model generalization and reduce overfitting, data augmentation techniques were applied exclusively to the training set after the dataset splitting process. The dataset was first divided into training (80%), validation (10%), and test (10%) subsets using a stratified approach. Subsequently, augmentation was performed only on the training subset, while the validation and test sets were kept completely unchanged to ensure unbiased model evaluation and to eliminate any risk of data leakage.

The augmentation pipeline included random horizontal and vertical flipping, rotation (± 20°), width and height shifting (up to 10%), zooming (range: 0.9–1.1), and random brightness and contrast adjustments. These transformations were applied probabilistically in an online manner during training, allowing the model to encounter diverse variations of the input data at each epoch.

This approach was designed to simulate real-world variability in histopathological images, such as differences in staining, orientation, and scale, thereby improving the robustness and generalization capability of the models.

### Deep learning models used

In order to maximize classification performance, five different deep learning architectures were designed and experimentally tested in this study. First, the VGG16 architecture was preferred. This model is a classical CNN architecture that stands out with its simple and deep structure, which gradually learns the spatial hierarchy within the image by using sequential small (3 × 3) convolution filters [8]. To reduce the gradient loss problem associated with deeper structures, the ResNet50 model was used. ResNet50 provides more stable and effective learning by preserving information flow in deep networks through residual connections [9]. In order to support multi-scale feature extraction, the InceptionV3 architecture was also included in the study. This model applies different filter sizes in parallel at the same layer level, allowing both fine and large-scale features to be captured simultaneously and providing advantages in terms of computational efficiency [10]. As a more modern approach, the EfficientNetV2S model was used. This architecture aims to obtain high classification accuracy with fewer parameters by scaling the depth, width, and input resolution of the network in a balanced manner [11]. Finally, the ConvNeXt-Tiny model was evaluated. Inspired by the design principles of Vision Transformer (ViT), this architecture aims to achieve “transformer-level” performance by reorganizing traditional CNN structures with modern architectural optimizations [12]. Details regarding the number of parameters and sizes of the models used in the study are presented in Table 1. Training of all models was performed using the Keras (Keras Core) library with the PyTorch backend.

**Table 1:** Parameter count and dimensionality information of models used for deep learning

Model	Parameter count	Size
VGG16	16.03 M	73.05 MB
ResNet50	28.05 M	145.13 MB
InceptionV3	26.27 M	138.58 MB
EfficientNetV2S	23.22 M	115.08 MB
ConvNeXt-Tiny	29.66 M	130.80 MB

### Model architecture

**Fig. 1:**
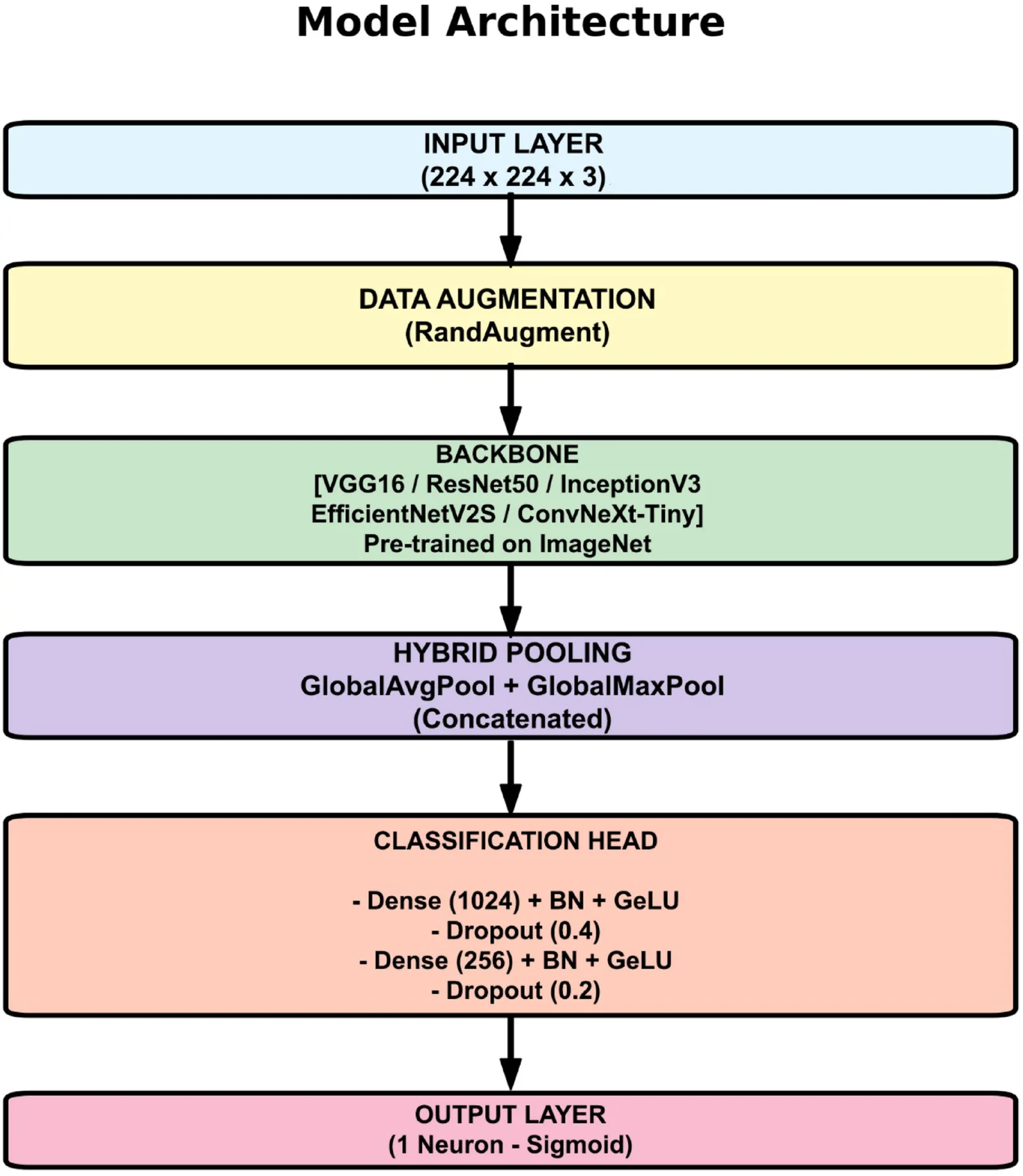
Model architecture. The classification head consists of two fully connected layers with GeLU activation functions, followed by a final output layer with a single neuron and sigmoid activation for binary classification

The model architecture is schematized in Fig. 1. In the model used in this study, pretrained ImageNet weights were used as feature extractors in the backbone parts, and only the custom classifier head was trained. The same classifier head was used for each model so that the comparison could be made in an unbiased manner.

The information coming from the feature extractor was passed through a hybrid pooling layer, where the outputs of Global Average Pooling and Global Max Pooling were combined and transferred to the next stage. This approach was preferred in order to better capture the most important features in the image.

In the classification layer, there are two main fully connected layers with 1024 and 256 neurons. Batch Normalization and GeLU activation were applied to the outputs of these layers in order to provide standardization, increase training stability, and improve performance through learning nonlinear features.

In addition, dropout rates of 40% and 20% were applied between these layers to prevent overfitting. As the output layer, a final layer with 1 neuron and a Sigmoid activation function was added.

### Model training

All models were trained using the same methodology. AdamW was selected as the optimizer and the learning rate was set to 0.0002. BinaryFocalCrossentropy was selected as the loss function and the label_smoothing parameter was set to 0.1.

During training, Accuracy and ROC-AUC were monitored as evaluation metrics. Training was set for 150 epochs. However, if there was no decrease in the loss value for 11 epochs, the training was stopped and the weights that achieved the best ROC-AUC score were restored.

Afterward, the best weights were saved and these weights were used for testing. Model trainings were performed separately on both the non-augmented original dataset and the augmented dataset.

### Classification threshold

For binary classification, a threshold value of 0.5 was used to convert predicted probabilities into class labels. Predictions with probabilities ≥ 0.5 were classified as OSCC, while those below 0.5 were classified as normal tissue.

### Statistical analysis and evaluation of model performances

In this study, the classification performances of the developed deep learning models were evaluated using Accuracy, Precision, Recall (Sensitivity), Specificity, F1-Score, and ROC-AUC metrics. All metrics were calculated based on the confusion matrix values obtained on the test dataset. All performance metrics were calculated on a strictly independent test set with no overlap with the training or validation data, ensuring an unbiased evaluation of model performance.

The confusion matrix consists of four main components: true positive (TP), true negative (TN), false positive (FP), and false negative (FN). Accuracy expresses the proportion of correctly classified samples among all samples and is calculated using the formula *(TP + TN)/(TP + TN+FP + FN).*

Precision shows how many of the samples predicted as positive by the model are actually positive and is calculated using the formula *TP/(TP + FP)*. This metric is particularly important in situations where false positive results are clinically significant.

Recall (Sensitivity) shows how many of the actual positive samples are correctly detected and is calculated using the formula *TP/(TP + FN).* Specificity expresses the rate of correctly classified true negative samples and is calculated using the formula *TN/(TN + FP).*

The F1-Score represents the harmonic mean of Precision and Recall and is calculated using the formula *2×(Precision×Recall)/(Precision+Recall).*

The ROC curve (Receiver Operating Characteristic) shows the relationship between sensitivity and 1-specificity at different threshold values. ROC-AUC (Area Under the Curve) represents the area under this curve and is accepted as a general indicator of the model’s ability to distinguish between classes. A ROC-AUC value approaching 1 indicates that the model has high discriminative performance.

To provide a more robust evaluation of model performance variability, bootstrap-based 95% confidence intervals (CIs) were additionally calculated for the main performance metrics using repeated resampling of the independent test set. Bootstrap resampling was performed with replacement over 1000 iterations. The resulting confidence intervals were used to assess the stability and uncertainty of the reported model performances.

In order to increase the interpretability of the model’s decision-making process, Grad-CAM (Gradient-weighted Class Activation Mapping) was used to generate heat maps. Grad-CAM determines the image regions that are most effective on the prediction by using gradient information from the last convolution layer of the model and shows these regions as a heat map on the image. The Grad-CAM analysis was performed as a qualitative visualization approach, and no quantitative overlap analysis with expert annotations was conducted in the present study.

With this method, it was possible to analyze which histopathological areas the model focused on when making an OSCC diagnosis. Thus, it was evaluated whether the decision-making process was based on clinically meaningful tissue regions. If the obtained heat maps show that the model concentrates on tumoral areas, this supports that the model outputs are biologically and pathologically consistent.

## Results

**Fig. 2:**
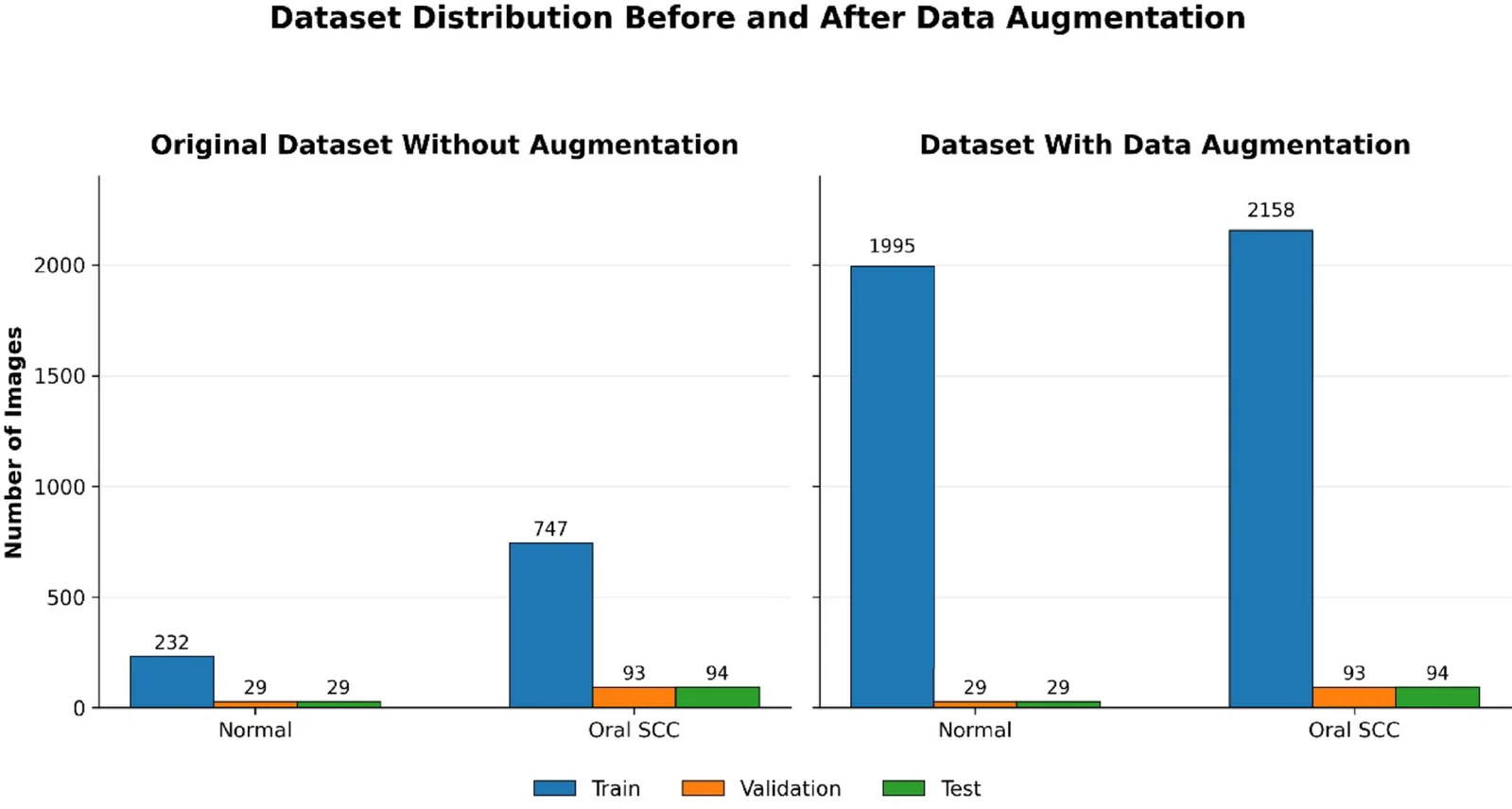
The distribution of OSCC and normal tissue in the training, validation, and test datasets

In the dataset without augmentation, a total of 1224 histopathological sections were used, with 979 images for training, 122 images for validation, and 123 images for the test dataset. In the augmented setting, data augmentation was applied only to the training set, increasing the effective number of training samples, while the validation (*n* = 122) and test (*n* = 123) sets remained unchanged. As a result, the training set was expanded through augmentation, whereas validation and test sets preserved their original distributions for unbiased evaluation. The distribution of OSCC and normal tissue in the training, validation, and test datasets is shown in Fig. 2. The classification results of the models were calculated using Accuracy, Precision, Recall, Specificity, F1-Score, and ROC-AUC metrics and are presented in Table 2. Bootstrap-based confidence interval analysis demonstrated that the performance estimates of the evaluated architectures showed a certain degree of overlap, particularly among high-performing models. Although ResNet50 achieved the highest overall performance metrics in the augmented dataset, the observed differences should be interpreted cautiously due to the relatively limited test sample size.

**Table 2:** Performance metrics of models trained on the original dataset and the augmented dataset

Model	Accuracy (95% CI)	Precision (95% CI)	Recall (95% CI)	Specificity (95% CI)	F1-Score (95% CI)	ROC-AUC (95% CI)
Model trained with original dataset without augmentation						
VGG16	0.83 (0.77–0.89)	0.88 (0.83–0.93)	0.90 (0.85–0.95)	0.62 (0.48–0.75)	0.89 (0.84–0.93)	0.76 (0.66–0.84)
ResNet50	0.81 (0.74–0.87)	0.88 (0.82–0.93)	0.87 (0.81–0.92)	0.62 (0.48–0.75)	0.87 (0.82–0.92)	0.74 (0.64–0.83)
InceptionV3	0.76 (0.68–0.83)	0.88 (0.81–0.93)	0.79 (0.71–0.87)	0.65 (0.52–0.78)	0.83 (0.76–0.89)	0.72 (0.62–0.81)
EfficientNetV2S	0.80 (0.73–0.86)	0.86 (0.80–0.92)	0.88 (0.82–0.93)	0.55 (0.41–0.69)	0.87 (0.81–0.92)	0.71 (0.61–0.80)
ConvNeXt-Tiny	0.83 (0.77–0.89)	0.88 (0.83–0.93)	0.90 (0.85–0.95)	0.62 (0.48–0.75)	0.89 (0.84–0.93)	0.76 (0.66–0.84)
Model trained with augmented dataset						
VGG16	0.86 (0.81–0.92)	0.89 (0.85–0.94)	0.93 (0.89–0.97)	0.65 (0.52–0.78)	0.91 (0.87–0.95)	0.91 (0.85–0.96)
ResNet50	0.91 (0.86–0.96)	0.94 (0.90–0.98)	0.94 (0.90–0.98)	0.82 (0.71–0.92)	0.94 (0.90–0.98)	0.88 (0.81–0.94)
InceptionV3	0.80 (0.73–0.86)	0.91 (0.85–0.96)	0.81 (0.74–0.89)	0.75 (0.63–0.86)	0.86 (0.79–0.92)	0.78 (0.69–0.87)
EfficientNetV2S	0.83 (0.77–0.89)	0.88 (0.82–0.93)	0.90 (0.85–0.95)	0.62 (0.48–0.75)	0.89 (0.84–0.93)	0.76 (0.66–0.84)
ConvNeXt-Tiny	0.87 (0.82–0.93)	0.91 (0.86–0.95)	0.92 (0.88–0.96)	0.72 (0.60–0.83)	0.92 (0.87–0.96)	0.82 (0.74–0.90)

On the original dataset, VGG16 and ConvNeXt-Tiny achieved the highest accuracy (0.83), followed by ResNet50 (0.81), EfficientNetV2S (0.80), and InceptionV3 (0.76). In the same dataset, the Precision values were calculated as 0.88 for VGG16 and ConvNeXt-Tiny, 0.88 for ResNet50, 0.88 for InceptionV3, and 0.86 for EfficientNetV2S. The Recall values were found to be 0.90 for VGG16 and ConvNeXt-Tiny, 0.88 for EfficientNetV2S, 0.87 for ResNet50, and 0.79 for InceptionV3. The Specificity values were generally lower and were calculated as 0.62 for VGG16, ResNet50, and ConvNeXt-Tiny, 0.65 for InceptionV3, and 0.55 for EfficientNetV2S. The F1-Score values were obtained as 0.89 for VGG16 and ConvNeXt-Tiny, 0.87 for ResNet50, 0.87 for EfficientNetV2S, and 0.83 for InceptionV3. The ROC-AUC values were calculated as 0.76 for VGG16, 0.74 for ResNet50, 0.72 for InceptionV3, 0.71 for EfficientNetV2S, and 0.76 for ConvNeXt-Tiny.

Data augmentation improved the performance of all models. In the augmented dataset, the Accuracy values were obtained as 0.86 for VGG16, 0.91 for ResNet50, 0.80 for InceptionV3, 0.83 for EfficientNetV2S, and 0.87 for ConvNeXt-Tiny. The Precision values were calculated as 0.89, 0.94, 0.91, 0.88, and 0.91, respectively. The Recall values were found as 0.93 for VGG16, 0.94 for ResNet50, 0.81 for InceptionV3, 0.90 for EfficientNetV2S and 0.92 for ConvNeXt-Tiny. The Specificity values were calculated as 0.65 for VGG16, 0.82 for ResNet50, 0.75 for InceptionV3, 0.62 for EfficientNetV2S, and 0.72 for ConvNeXt-Tiny. The F1-Score values were obtained as 0.91 for VGG16, 0.94 for ResNet50, 0.86 for InceptionV3, 0.89 for EfficientNetV2S, and 0.92 for ConvNeXt-Tiny. The ROC-AUC values were found as 0.91, 0.88, 0.78, 0.76, and 0.82, respectively.

Comparison of the two datasets demonstrated that data augmentation particularly improved accuracy, specificity, and ROC-AUC values. In particular, the ResNet50 model demonstrated the highest performance with 0.91 accuracy and 0.88 ROC-AUC in the augmented dataset. In addition, the VGG16 and ConvNeXt-Tiny models also showed noticeable performance improvements after augmentation.

**Fig. 3:**
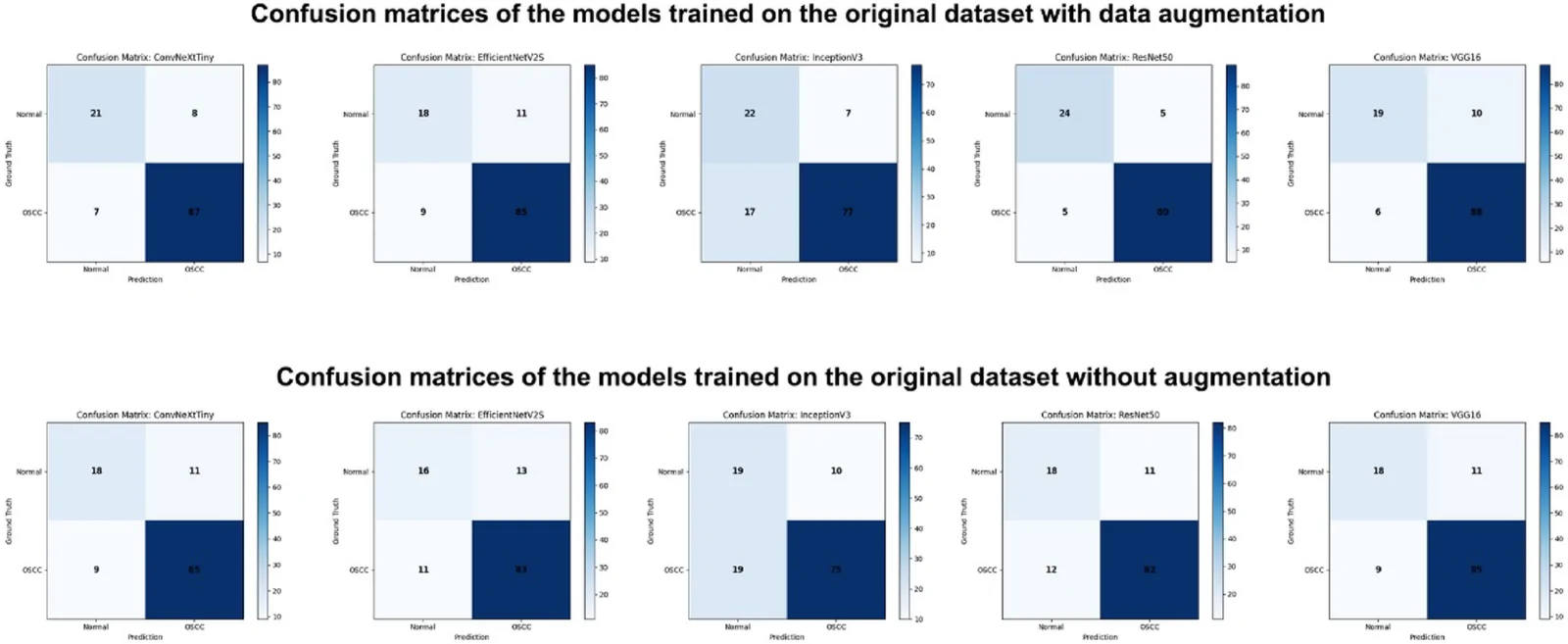
The confusion matrices generated for each model

The confusion matrices generated for each model are shown in Fig. 3. In these figures, the numbers of TP, TN, FP, and FN classifications obtained on the test dataset are presented for each model. The confusion matrices numerically show the distribution of correct and incorrect predictions made by the models in distinguishing OSCC and normal tissue.

**Fig. 4:**
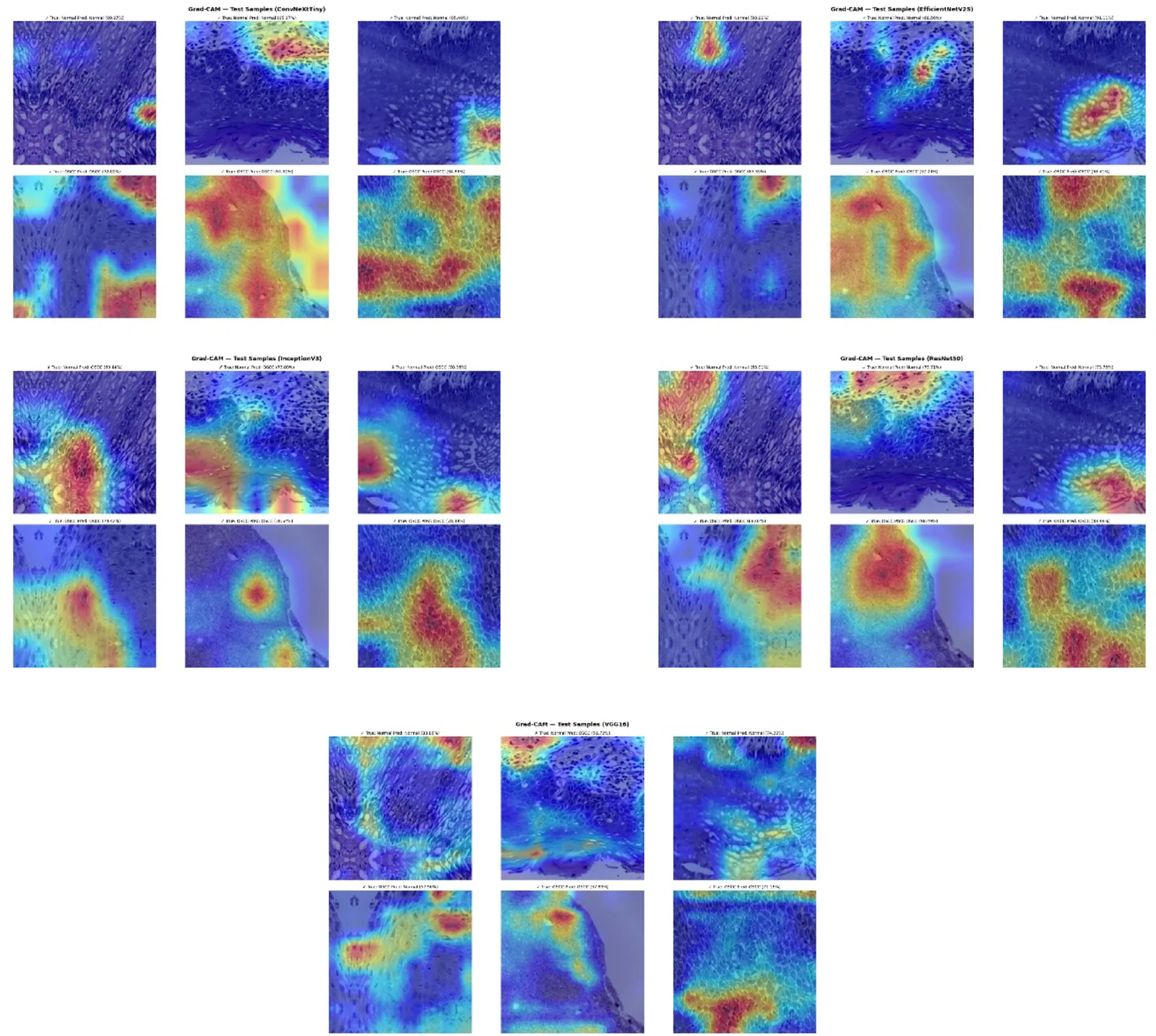
Representative Grad-CAM visualizations generated for the evaluated CNN architectures. Warmer color regions indicate areas contributing more strongly to the model predictions for OSCC. High-resolution versions of the Grad-CAM visualizations are additionally provided in the supplementary material to facilitate clearer assessment of the correspondence between histopathological structures and activation patterns

The class activation maps obtained using the Grad-CAM method are presented in Fig. 4 and Supplementary Material 1. These heat maps show which regions of the image the model assigns greater weight to during the prediction process. In the samples classified as OSCC for each model, it was observed that the activation intensity was concentrated in specific histopathological areas. Figure 4 visually demonstrates the regions influencing model predictions.

## Discussion

This study provides a systematic comparison of different CNN architectures for automated classification of OSCC histopathological images within a unified experimental framework. Whole-slide images (WSIs) and patch-based analyses have been reported to capture morphological patterns beyond the visual perception of pathologists, thereby supporting diagnosis, grading, and even prognostic inference; however, barriers such as data standardization, validation design, and real-world distribution shifts (scanner/color/laboratory variation) remain major limitations [16–18]. In the context of oral cancer, recent studies reviewing deep learning applications across different data modalities (clinical images, histopathology, genomics, etc.) emphasize that insufficient data quality and the lack of external validation may inflate reported performance metrics [19, 20]. In this context, our study attempted to provide a methodologically transparent framework by performing multi-backbone comparisons using the same dataset and the same classification head. A major strength of this study is the controlled experimental design, in which all CNN architectures were evaluated under identical conditions using a leakage-free dataset and a fixed test set. This approach allows for a more reliable comparison than many previous studies and provides practical insights into model selection for clinical applications.

According to the findings of our study, the relatively low specificity values observed in some models without augmentation are consistent with patterns frequently encountered in histopathology, such as class imbalance, patch selection bias, and tissue heterogeneity. The current WSI literature emphasizes that in classifiers developed particularly at the patch level, the risk of false positives may increase due to staining variations, artifacts, and morphological similarities of non-neoplastic epithelium, and that this situation should be managed through threshold optimization as well as data diversity and augmentation strategies [16–18]. In oral cancer histopathology, it has also been reported that there may be significant differences in performance and interpretability between classical single-instance learning (SIL) and multiple-instance learning (MIL), which is more suitable for patient/slide-level inference; MIL can produce more consistent interpretations especially in scenarios where the ground truth is available at the patient level [21]. Therefore, the improvement in specificity after augmentation likely reflects more robust learning against staining and scale variations. The relatively lower specificity values observed in some models may also be associated with the morphological similarity between reactive/non-neoplastic epithelial regions and early tumoral changes in histopathological images. In addition, the class distribution and limited diversity of negative samples within the dataset may have contributed to an increased rate of false-positive predictions. These factors are commonly encountered challenges in patch-based histopathological classification tasks and may partially explain the imbalance between sensitivity and specificity observed in certain architectures.

In the comparison between models, the fact that ResNet50 achieved the highest overall classification performance in the augmented scenario is consistent with the trend in the current oral pathology artificial intelligence literature: in practice, “moderately deep, well-optimized residual” backbones often provide a strong balance between learnability and generalizability in small-to-medium-scale histopathology datasets. Indeed, recent studies show that systems developed for automatic diagnosis and grading of OSCC histopathology images are often built around the ResNet family or similar strong CNN backbones [22, 23]. However, it has also been demonstrated that when the goal is not only binary classification but also multi-class grading or subtype differentiation in oral cancer, performance (especially accuracy) may decrease, and that dataset size and label reliability play a decisive role in this decline [23, 24]. When interpreted together with the broad improvement of metrics before and after augmentation observed in our study, this finding further strengthens the importance of data diversity in heterogeneous tumors such as OSCC.

The strong performance observed in our findings for modern architectures (EfficientNet family and next-generation optimized CNNs) is also consistent with current studies. It has been reported that EfficientNet-based approaches in OSCC pathological images can achieve high accuracy and sensitivity, and that lightweight architectures have the potential to reduce computational costs in clinical applications [25, 26]. Moreover, recent studies focusing on explainability emphasize that beyond raw performance, presenting the model’s decision rationale through clinically meaningful regions (e.g., epithelial dysplasia or areas surrounding tumor islands) increases the translational value of such systems [22–24]. In this context, the Grad-CAM analysis presented in our study showing that the model focuses on areas associated with OSCC constitutes an important argument in the discussion, supporting that the high ROC-AUC values reported are accompanied by a “pathologically plausible” attention mechanism.

On the explainability (XAI) side, current digital pathology sources particularly emphasize the difference between “generating a heatmap” and “producing clinically verifiable explanations”: although Grad-CAM and similar methods provide a good starting point, the stability of explanations should be tested across different stainings, scanners, and external datasets [16–18]. In the context of oral cancer, Grad-CAM–based multi-stage grading or explainable multi-class approaches have been reported with the aim of increasing objectivity, particularly when labels are created based on the evaluation of two pathologists [24, 27]. Therefore, in future studies in the same field, blind evaluation of Grad-CAM maps by pathology experts (e.g., “overlap rate with tumoral epithelium/keratinization regions”) and stability analysis using external datasets may provide a strong extension in terms of clinical confidence and publication impact. However, the Grad-CAM analysis in the present study was evaluated qualitatively, and no quantitative explainability validation was performed. Specifically, no annotation overlap analysis, region-level quantitative assessment, or expert-guided evaluation by pathologists was conducted to measure the correspondence between highlighted regions and histopathologically relevant tumoral structures. Therefore, although the generated heatmaps visually demonstrated biologically plausible attention patterns, the interpretability findings should be considered exploratory rather than definitive. Future studies incorporating quantitative explainability metrics, expert annotation overlap analyses, and blinded pathologist-based evaluations may provide stronger evidence regarding the reliability, consistency, and clinical relevance of the highlighted regions.

In OSCC histopathology, not only classification but also semantic segmentation of tumoral regions may enable improvements in both interpretability and the reduction of false positive/negative predictions. Recent studies reporting comprehensive evaluations of semantic segmentation in OSCC histopathological images show that the success of tissue boundary detection may vary between different architectures and that region-based outputs may provide practical advantages in clinical use [28]. In addition, within the WSI context, attention/MIL frameworks have been reported to quantitatively present which patches contribute to the final decision while generating slide-level predictions, thereby contributing to interpretability and traceability [21, 29]. Although the approach presented in this study produced very strong metrics in patch-based classification, supporting it in future research with WSI/MIL frameworks or segmentation methods may provide a translation closer to real-world pathology workflows. In addition, since the present study was conducted using image/patch-based classification rather than whole-slide image (WSI) or patient-level analysis, the reported performance metrics should be interpreted within this experimental scope. Although patch-based approaches are valuable for identifying localized histopathological patterns, direct translation to slide-level or patient-level diagnosis remains limited. Future studies integrating WSI-based pipelines and multiple instance learning (MIL) frameworks may provide more clinically applicable and robust diagnostic systems by enabling patient-level inference and improved contextual analysis. In real-world pathology workflows, diagnostic decisions are typically established at the whole-slide or patient level rather than from isolated image patches. Therefore, although the high performance metrics obtained in the present study demonstrate the capability of CNN-based models to identify localized histopathological patterns associated with OSCC, these findings should not be directly interpreted as equivalent to fully automated clinical diagnostic performance. The transition from patch-level classification to clinically deployable slide-level systems remains a major challenge in computational pathology due to issues such as tissue heterogeneity, contextual dependency, and aggregation of patch-level predictions. Consequently, further studies integrating whole-slide image analysis, multiple-instance learning frameworks, and patient-level validation are required before clinical implementation can be considered.

Studies performing artificial intelligence-based classification on different histopathological section/WSI datasets in the literature generally report very high performance metrics, presenting a picture consistent with the strong ROC-AUC and accuracy values obtained after augmentation in this study. For example, in breast histopathology, a study using MobileNetV3 reported 0.98 precision, 0.97 recall, and 0.98 F1 for benign–malignant classification, while class-dependent variations such as 0.95 accuracy for ductal carcinoma were also observed [30]. In colorectal histopathology, the Color-CADx approach reported 99.3% accuracy on the NCT-CRC-HE-100 K dataset and 96.8% accuracy on the Kather_texture_2016_image_tiles dataset [31]. In cervical histopathology (SipakMed), the proposed CCanNet model reported 98.53% accuracy in a multi-model comparison, demonstrating that high performance can also be achieved with lightweight architectures [32]. In oral cancer images (MOD and OCI datasets), the lightweight and explainable LWENet approach was reported with values such as 97.86% accuracy and AUC = 0.9897 on MOD and 98.99% accuracy on OCI [22]. In endometrial cancer histopathology, the ECgMLP model reported 99.26% accuracy for multi-class categories [33]. At the WSI level, the DG-SSM-MIL study, a modern MIL-based framework, reported AUC = 0.957 (CAMELYON16) with ResNet-50 features and competitive AUC values across different public datasets, providing quantitative evidence for the generalizability of these methods across different tissues [34]. In multi-class colorectal category detection, the report of 85.04% overall accuracy and macro ROC-AUC = 0.9850 using ResNet-34 indicates that although accuracy may decrease as the number of classes and morphological diversity increase, discriminative power (AUC) can remain high [35]. 

This study has several limitations. (i) Dependence on a single open dataset, (ii) uncertainty in transferring patch-level labels to slide/patient-level inference, and (iii) the lack of external validation against staining and scanner variations represent the most critical issues. Although the present findings demonstrate promising diagnostic performance under controlled experimental conditions, the use of a single-source dataset may limit the generalizability of the models across different clinical environments. In digital pathology applications, model performance may be substantially influenced by variations in staining protocols, slide preparation procedures, scanner hardware, image resolution, and institutional workflow differences. Since the present study relied exclusively on a single publicly available dataset obtained under relatively homogeneous conditions, the reported performance metrics may not fully reflect real-world performance across different clinical centers. Therefore, external validation using independent multicenter datasets is essential to evaluate the robustness, reproducibility, and domain generalizability of the proposed models prior to potential clinical implementation. Therefore, future studies should include external and multi-center validation cohorts obtained from different institutions, scanners, and staining protocols to further evaluate the robustness, reproducibility, and clinical applicability of the proposed models. Current reviews and framework papers in digital pathology emphasize the importance of external validation across centers, reporting standards, data governance, and the complete presentation of methodological details for clinical integration [16–18, 36]. Therefore, in future studies, the inclusion of multi-center datasets, stain normalization/domain adaptation methods, and patient-level validation (preferably with MIL/WSI approaches) may increase the clinical translatability of the high performance obtained. Such external validation strategies are particularly important for minimizing domain shift effects and ensuring that model performance remains stable across heterogeneous clinical environments.

## Conclusion

This study comparatively evaluated the performances of different deep learning architectures for patch-based classification of OSCC histopathological images within a controlled experimental framework. The findings demonstrate that deep learning–based approaches can effectively identify histopathological patterns associated with OSCC and that data augmentation substantially improves model robustness and classification performance. However, since the present study was conducted at the patch level rather than the whole-slide or patient level, the reported performance metrics should be interpreted within this experimental scope. Future studies incorporating external multicenter validation datasets obtained from different institutions, scanners, and staining protocols are necessary to evaluate the robustness, reproducibility, and real-world clinical applicability of these systems. In addition, future studies integrating quantitative explainability analyses and expert-guided validation frameworks may further improve the interpretability and clinical reliability of AI-assisted diagnostic systems.

## Supplementary Information

Below is the link to the electronic supplementary material.

Supplementary Material 1

## Data Availability

The datasets used and/or analyzed during the current study are available from the corresponding author on reasonable request.

## Abbreviations

CNN: Convolutional Neural Network
OSCC: Oral Squamous Cell Carcinoma
ROC: Receiver Operating Characteristic
ROC- AUC: Area Under the Receiver Operating Characteristic Curve
TP: True Positive
TN: True Negative
FP: False Positive
FN: False Negative
WSI: Whole Slide Image
SIL: Single Instance Learning
MIL: Multiple Instance Learning
XAI: Explainable Artificial Intelligence
Grad-CAM: Gradient—weighted Class Activation Mapping
