# Supplementary material for: Deep learning–based automated detection of oral squamous cell carcinoma in histopathological images: a comparative study of five CNN architectures
Source: Odontology. 2026 Jun 15;114(4):2742–53. doi: 10.1007/s10266-026-01448-7 (PMC13630891; doi:10.1007/s10266-026-01448-7)
Supplement: Supplementary file 1 [file 10266_2026_1448_MOESM1_ESM.pdf]

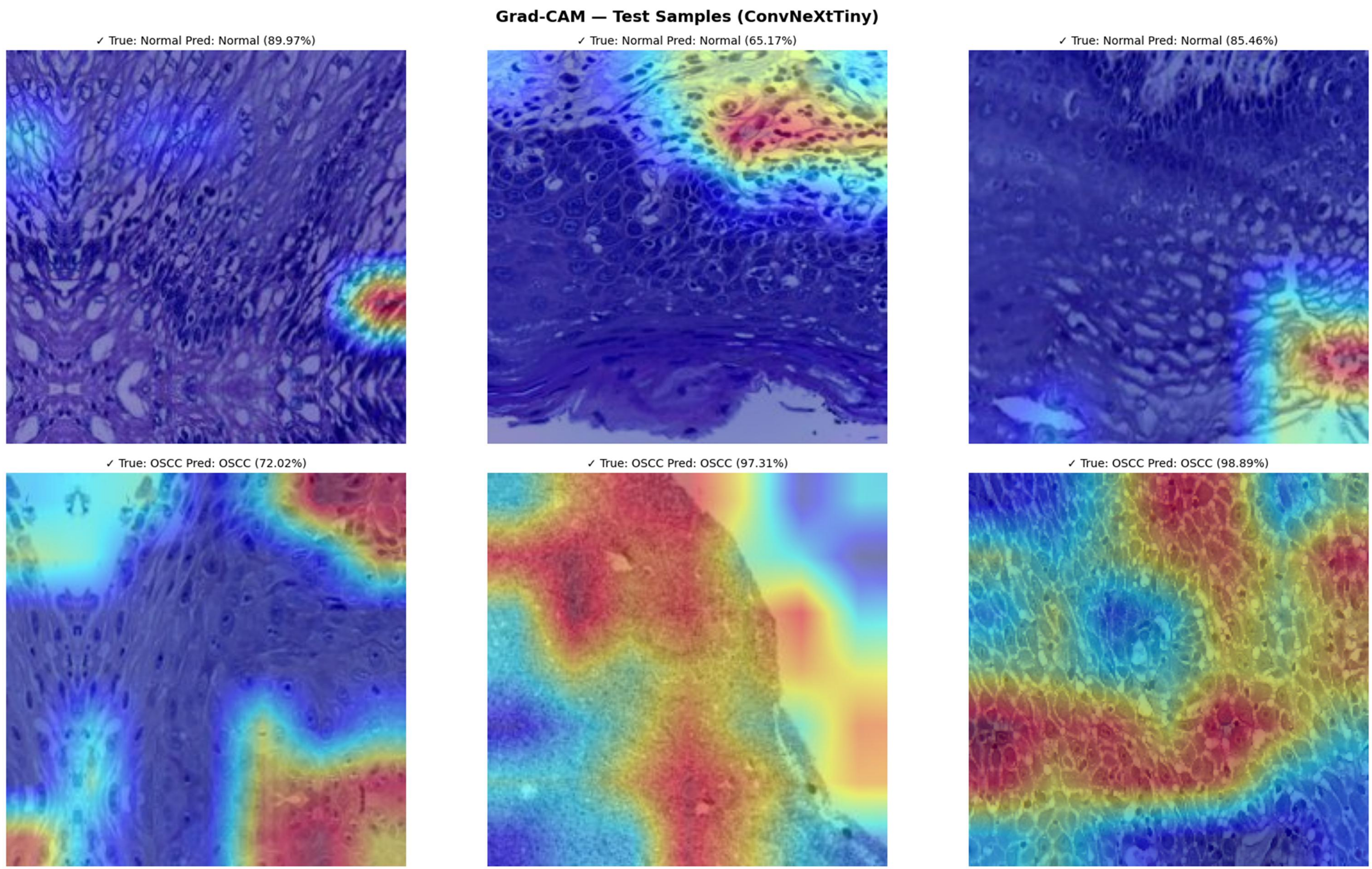
Grad-CAM — Test Samples (ConvNeXtTiny)
✓ True: Normal Pred: Normal (89.97%)
✓ True: Normal Pred: Normal (65.17%)
✓ True: Normal Pred: Normal (85.46%)
✓ True: OSCC Pred: OSCC (72.02%)
✓ True: OSCC Pred: OSCC (97.31%)
✓ True: OSCC Pred: OSCC (98.89%)

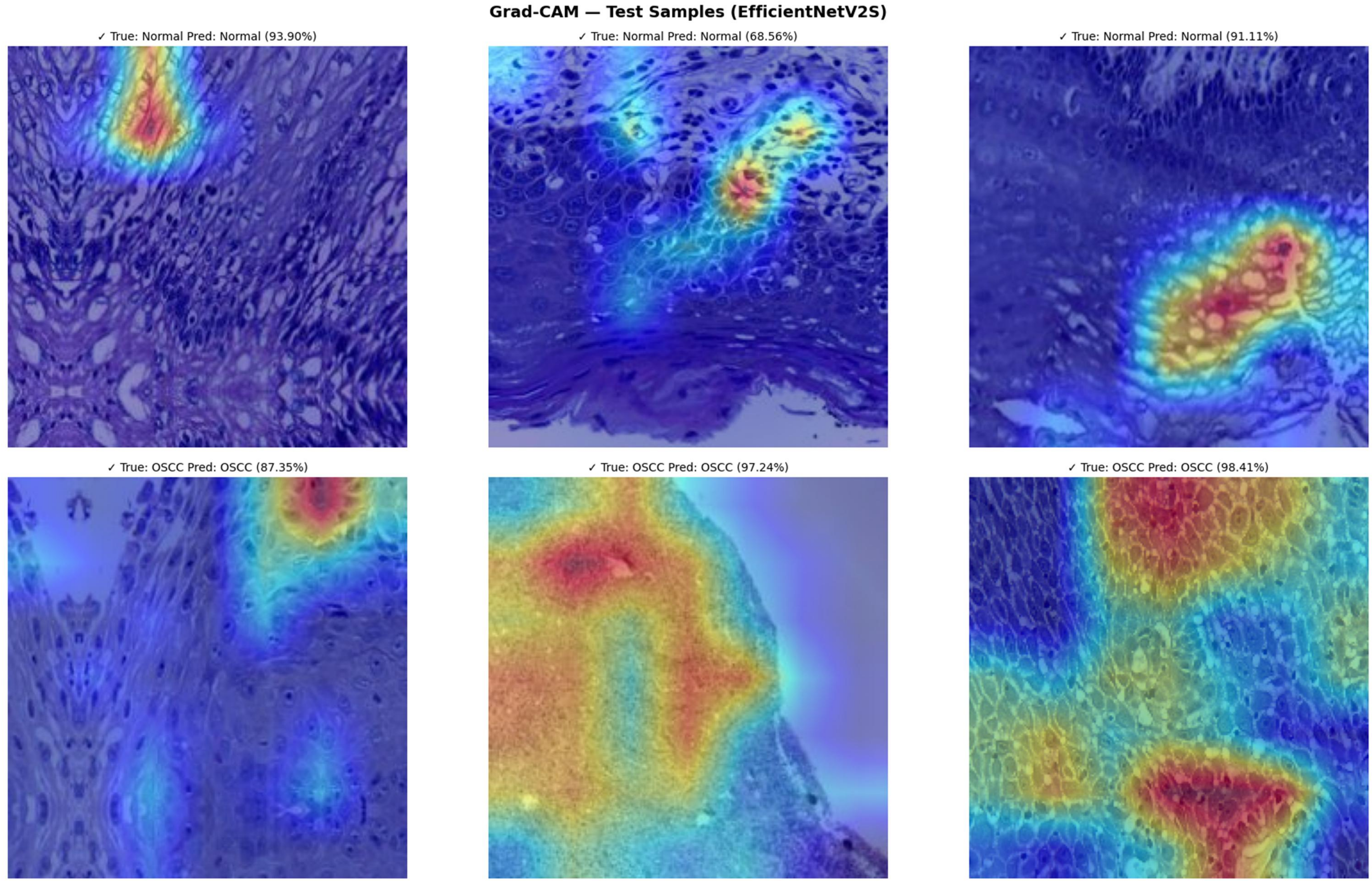
Grad-CAM — Test Samples (EfficientNetV2S)
✓ True: Normal Pred: Normal (93.90%)
✓ True: Normal Pred: Normal (68.56%)
✓ True: Normal Pred: Normal (91.11%)
✓ True: OSCC Pred: OSCC (87.35%)
✓ True: OSCC Pred: OSCC (97.24%)
✓ True: OSCC Pred: OSCC (98.41%)

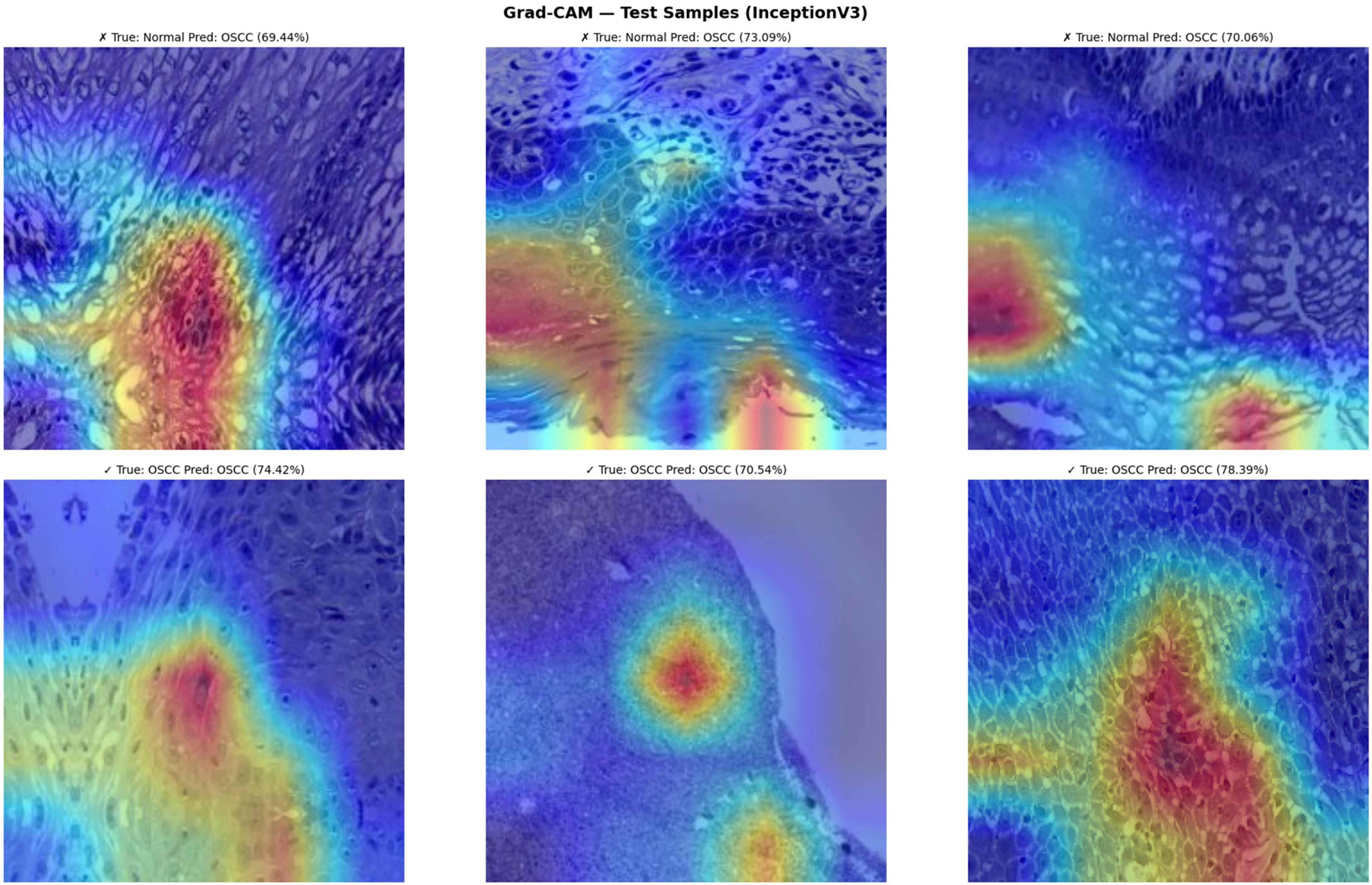
Grad-CAM — Test Samples (InceptionV3)
✗ True: Normal Pred: OSCC (69.44%)
✗ True: Normal Pred: OSCC (73.09%)
✗ True: Normal Pred: OSCC (70.06%)
✓ True: OSCC Pred: OSCC (74.42%)
✓ True: OSCC Pred: OSCC (70.54%)
✓ True: OSCC Pred: OSCC (78.39%)

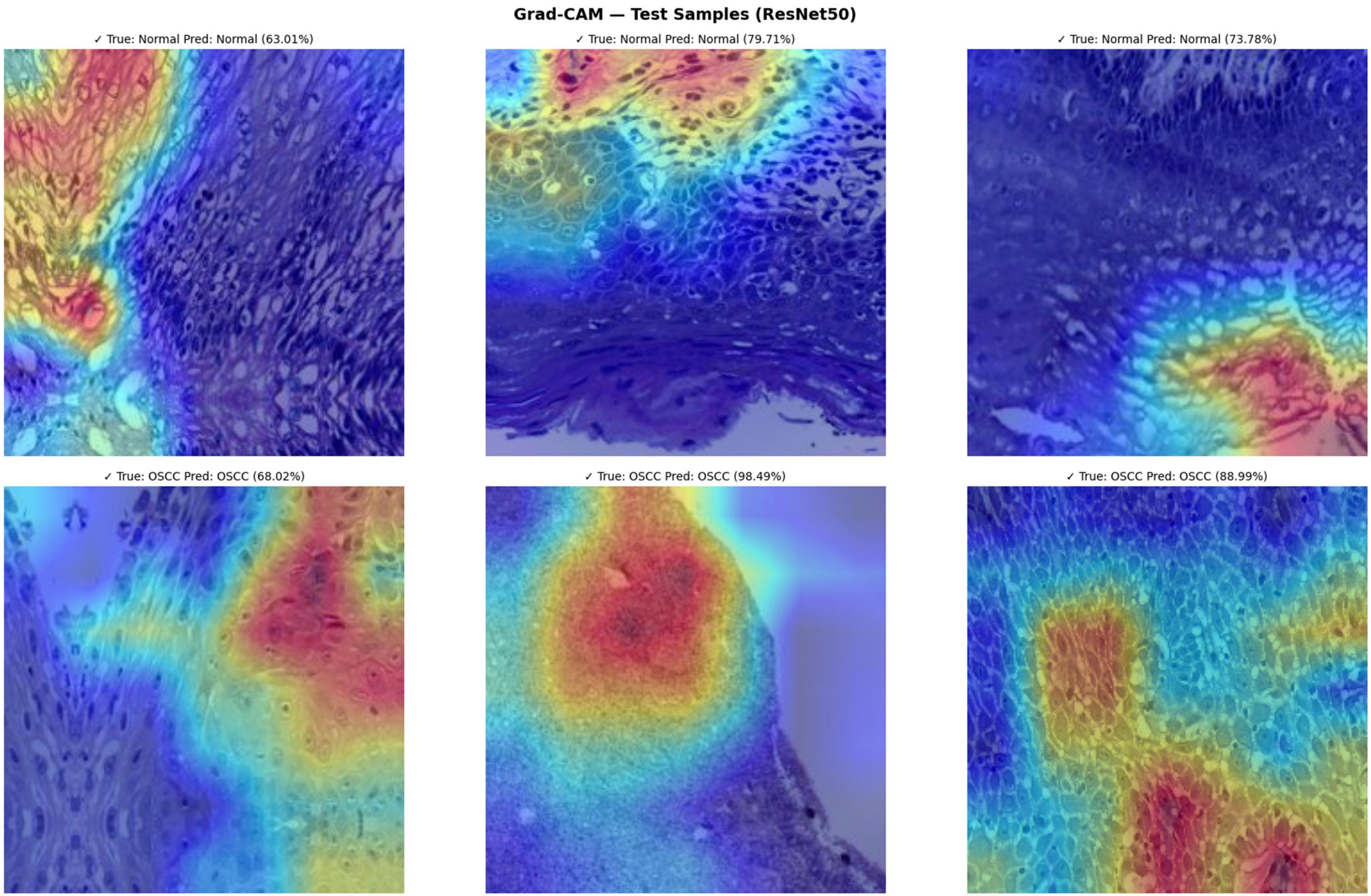
Grad-CAM — Test Samples (ResNet50)
✓ True: Normal Pred: Normal (63.01%)
✓ True: Normal Pred: Normal (79.71%)
✓ True: Normal Pred: Normal (73.78%)
✓ True: OSCC Pred: OSCC (68.02%)
✓ True: OSCC Pred: OSCC (98.49%)
✓ True: OSCC Pred: OSCC (88.99%)

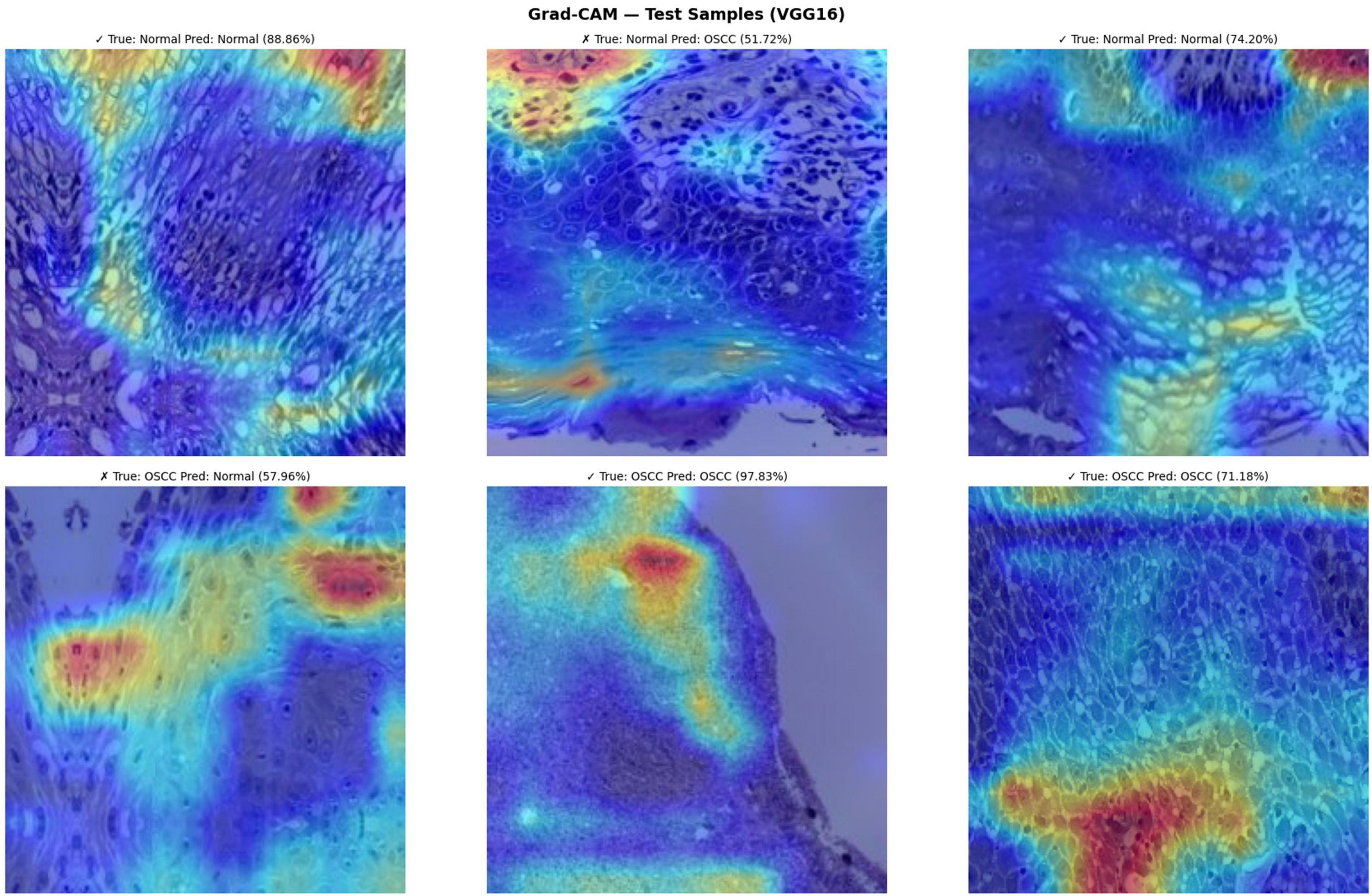
Grad-CAM — Test Samples (VGG16)
✓ True: Normal Pred: Normal (88.86%)
✗ True: Normal Pred: OSCC (51.72%)
✓ True: Normal Pred: Normal (74.20%)
✗ True: OSCC Pred: Normal (57.96%)
✓ True: OSCC Pred: OSCC (97.83%)
✓ True: OSCC Pred: OSCC (71.18%)
